# Exploring the causal relationship between the immune cell-inflammatory factor axis and lung cancer: a Mendelian randomization study

**DOI:** 10.3389/fonc.2024.1345765

**Published:** 2024-08-29

**Authors:** Lin Zhu, Zhi Jin

**Affiliations:** Department of Traditional Chinese Medicine, The Second Hospital of Shandong University, Jinan, China

**Keywords:** lung cancer, immune cells, inflammatory factors, causal relationship, Mendelian randomization

## Abstract

**Background:**

Lung cancer is a major health burden globally and smoking is a well-known risk factor. It has been observed that chronic inflammation contributes to lung cancer progression, with immune cells and inflammatory cytokines implicated in tumor development. Clarifying the causal links between these immune components and lung cancer could enhance prevention and therapy.

**Methods:**

We performed Mendelian randomization (MR) to explore causal connections between immune cells, inflammatory markers, and lung cancer risk, using genetic variants as instruments. Data from GWAS on these variables underpinned our MR analyses.

**Results:**

Our findings indicated an inverse association between some immune cells and lung cancer risk, implying that more immune cells might be protective. NK T cells (CD16-CD56) and myeloid cells (HLA DR+ on CD33dim HLA DR+ CD11b+) had an inverse correlation with lung cancer risk. Furthermore, a direct relationship was observed between inflammatory cytokines and these immune cells. In contrast, IL-18 was inversely associated with lung cancer, while IL-13 showed a direct correlation.

**Conclusion:**

The study underscores the role of immune and inflammatory factors in lung cancer. These insights could lead to new therapeutic strategies for combating lung cancer.

## 1 Introduction

Lung cancer, a malignant tumor originating from lung tissues, is among the most prevalent cancers globally and a leading cause of cancer-related mortality (1, 2). Every year, approximately 2.2 million new cases of lung cancer are diagnosed worldwide, with higher incidence rates in men than in women (3, 4). Smoking is the primary risk factor for lung cancer, and the risk of lung cancer is significantly higher in smokers (5). Inflammation plays a crucial role in the development of lung cancer, with persistent chronic inflammation closely associated with lung cancer occurrence, and smoking, air pollution, asbestos exposure, and other factors acting as causative factors (6, 7). These inflammatory stimuli induce chronic inflammatory responses in lung tissues, thus promoting tumor formation (8). Moreover, lung cancer tissues often exhibit increased infiltration of inflammatory cells such as macrophages, lymphocytes, and neutrophils, which release various inflammatory mediators that promote tumor growth, invasion, and metastasis (9, 10). The significant role of immune cells in lung cancer has been emphasized, as they can recognize and attack tumor cells, thus inhibiting tumor growth and spread (11). However, tumor cells can evade the attack of immune cells through various mechanisms, thus promoting tumor development and progression. Research indicates alterations in the number and function of immune cells in patients with lung cancer, including the presence of several macrophages and dendritic cells, which can recognize and attack tumor cells. Yet, tumor cells can escape immune cell attacks by secreting immunosuppressive factors that inhibit immune cell function (12, 13). Additionally, immune cells in patients with lung cancer may function abnormally. For example, they may suppress natural killer (NK) cells and T-lymphocytes, which leads to ineffective attacks on tumor cells (14). The abnormal function of these immune cells may be associated with the secretion of immunosuppressive factors secreted by tumor cells (15, 16). Therefore, observing immune cells in patients with lung cancer is crucial for understanding the mechanism underlying lung cancer development and designing effective immunotherapy strategies.

Mendelian randomization (MR) is a method in which genetic instruments are used to examine the causal relationship between modifiable exposures and outcomes (17, 18). It simulates the effects of randomized clinical trials using genetic variants as indicators of natural random assignment to evaluate the causal effect of exposure factors on outcomes (19). In our study, we used MR to investigate the relationship between immune cell infiltration in lung cancer and explore the role of inflammatory factors in this process.

## 2 Materials and methods

### 2.1 Study design

**Figure 1:**
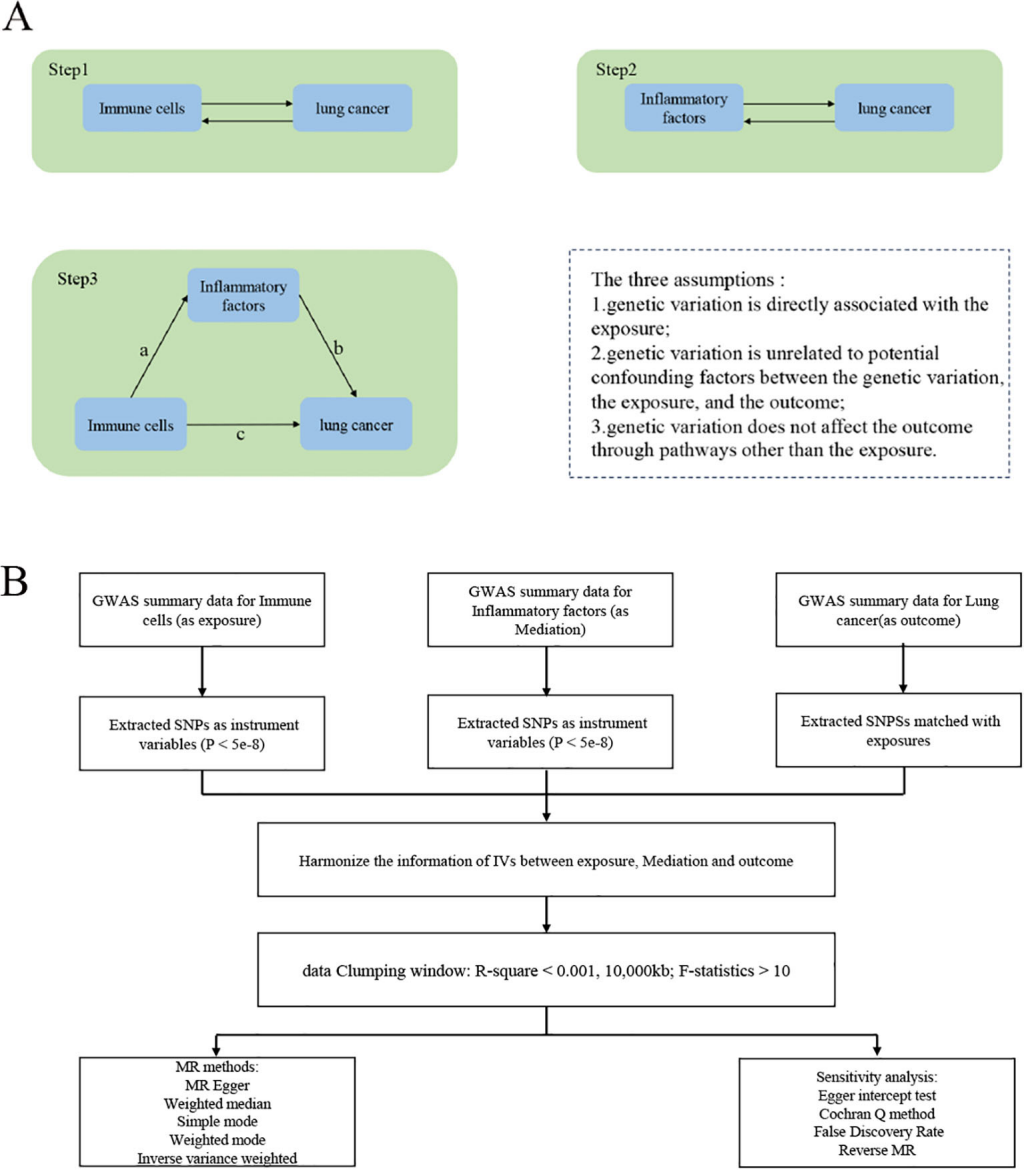
Study Overview. **(A)** The first step1: illustrates the causal effects of immune cells on lung cancer. The second step: illustrates the causal effects of inflammatory factors on lung cancer. The third step: represents intermediate analysis through the pathway of inflammatory factors from immune cells to lung cancer: Pathway c represents the overall impact of immune cells on Lung cancer; Pathway b represents the causal effects of inflammatory factors on Lung cancer; Pathway a represents the causal effects of immune cells on inflammatory factors. Use the two-step mesomeric effect (where a is the total effect of Immune cells on inflammatory factors, and b is the effect of inflammatory factors on lung cancer) and the product method (a × b) Direct effect = c−mesomeric effect. **(B)** The mechanic diagram of Mendelian Randomization Study.

The study was conducted in three steps (**Figure 1A**). Step 1: Analysis of the causal effects of immune cells on lung cancer; Step 2: Analysis of the causal effects of inflammatory factors on lung cancer; Step 3: Mediation analysis of inflammatory factors in the pathway from immune cells to lung cancer. MR uses genetic variation as a proxy for risk factors, and therefore, effective instrumental variables (IVs) must satisfy three key assumptions for causal inference: (1) genetic variation is directly associated with the exposure; (2) genetic variation is unrelated to potential confounders between the exposure and outcome; (3) genetic variation does not affect the outcome through pathways other than the exposure. The experimental flowchart is shown in (**Figure 1B**)

### 2.2 Data sources

#### 2.2.1 Source of immune cell data

The Genome-Wide Association Study (GWAS) data on immune cells in this study is derived from a research study that included individuals aged 18 to 102. Using a family-based cohort, 3,757 individuals from a general population of 6,602 individuals from the eastern coast of Sardinia with varying degrees of genetic relatedness underwent in-depth genetic and immune feature analysis, covering 539 immune features including cell counts, median fluorescence intensity (MFI) of cell surface antigens, and morphological parameters. These cell types emerge during embryonic development and hematopoietic stem cell transplantation. Through GWAS, the research team tested a large number of genetic variations and identified 122 independent significant signals, with 16 being rare in Europeans but more common in Sardinians. Additionally, 53 signals overlapping with previously reported disease-related signals were found, primarily related to autoimmune diseases. These results reveal the complex genetic regulation of immune cells, with highly selective effects on the risk of autoimmune diseases. The GWAS data stem from a study on genetic features of immune cells, identifying genetic variations associated with immune cell features through multiple independent tests. We selected 731 immune cell phenotypes from the study, including Absolute Count (AC) (n=118), Median Fluorescence Intensity (MFI) indicating surface antigen levels (n=389), Morphological Parameters (MP) (n=32), and Relative Cell Count (RC) (n=192). Specifically, MFI, AC, and RC features are derived from B cells, CDCs, mature T cells, monocytes, myeloid cells, TBNK (T, B, and NK cells), and Treg panels. MP features include CDC and TBNK panels (20, 21).

#### 2.2.2 Source of inflammatory factor data

The GWAS data on inflammatory factors is derived from a study aimed at identifying 27 genetic loci that influence the concentration of circulating cytokines and growth factors. The research identified genetic variations associated with various cytokines, with 15 loci showing expression trait quantitative trait loci in whole blood. The study results provide genetic tools to elucidate cytokine signaling and the causal role of upstream inflammation in immune-related and other chronic diseases (22).. To normalize the distribution of the 41 cytokines, a two-step inverse transformation was applied.

To test the univariable associations between 10.7 million genetic polymorphisms and the concentrations of 41 cytokines, we used an additive genetic model. This model considered adjustments for age, sex, body mass index, and the first ten genetic principal components.

#### 2.2.3 Source of lung cancer data

Lung cancer data were retrieved from International Lung Cancer Consortium for Lung Cancer Interdisciplinary Research (TRICL-ILCCO), which is an interdisciplinary research alliance comprising multiple international research teams studying the genetic and environmental factors that influence the onset and development of lung cancer. The primary objective of TRICL-ILCCO is to strengthen research on lung cancer and improve our understanding of lung cancer prevention, diagnosis, and treatment through collaboration and data sharing. The alliance is dedicated to the collection and integration of global large-scale lung cancer research data, including genomics epidemiological, clinical, and biological data, for comprehensive analysis and interpretation. We included 9298 samples and 7,024,138 SNPs.

### 2.3 Instrumental variable selection

We restricted the inclusion criteria for IVs to ensure the accuracy and effectiveness of the causal relationship between immune cells and lung cancer risk. First, in MR studies, only SNPs with P-values < 5e-08 were included as IVs for exposure and outcome. Second, using the TwoSampleMR R package, to mitigate the impact of linkage disequilibrium (LD) on the independence of instrumental variables (IVs), we employed a clustering distance window of 10,000 kb and calculated the LD independence correlation (r^2), consequently eliminating SNPs with r^2 < 0.01. Finally, we used the F-statistic to assess the strength of each SNP, which considers the magnitude and precision of genetic effects on traits. The formula used for F was as follows: F = R² (N − 2)/(1 − R²), where R² represents the proportion of trait variance explained by the SNP, and N is the sample size of the GWAS associated with the trait.

To estimate R², we used the formula R² = 2 × EAF × (1 − EAF) × β², where EAF represents the allele frequency of the SNP and β represents the estimated effect of the SNP on the trait. This formula allows us to estimate the proportion of variance in the trait explained by the SNP.

We excluded SNPs with an F-statistic less than 10, as an F-statistic greater than 10 indicates sufficient strength to ensure the validity of the SNPs.

### 2.4 Statistical analysis

MR is used to investigate causal relationships between a modifiable exposure and an outcome using genetic instruments. There are two key assumptions in MR. Assumption 1 states that the genetic instruments are associated with the exposure of interest, and assumption 2 states that any association between the instruments and the outcome is mediated by the exposure (23). To address these assumptions, five MR methods were used in the analysis. The ratio method involved obtaining individual SNP estimates by dividing the SNP’s effect on lung cancer by its corresponding effect on the biomarker. Standard errors were estimated assuming no measurement error. These estimates were then used for weighted analyses using other methods. Inverse variance weighting (IVW) is a commonly used method in MR (24, 25). It calculates the inverse variance weighted mean of ratio estimates from multiple instruments. In this method, all SNPs are assumed to be valid instruments, or any bias is considered to be balanced across the instruments. Both fixed and random effects IVW methods were used. Weighted generalized linear regression is similar to the IVW method but accounts for the correlation between genetic instruments. It is used with a conservative set of genetic instruments. The weighted median method calculates the median of the weighted empirical distribution function of individual SNP ratio estimates. This method provides a consistent effect estimate if more than 50% of the information is derived from valid SNPs (21, 26). In MR Egger regression, we conducted the weighted linear regression of SNPlung cancer against SNP biomarker effect estimates (27). It assumes that the horizontal pleiotropic effects and SNP exposure associations are not correlated. The intercept of MR Egger regression can be interpreted as a test for overall unbalanced horizontal pleiotropy. Both fixed and random effect versions of this method were used. By using these five MR methods, we attempted to minimize bias and obtain reliable estimates of the causal relationship between the modifiable exposure and the outcome of interest. Different causality analysis models were used in this study. Among them, the inverse-variance weighted (IVW) model and MR-Egger method (28)were used for analyzing samples with multiple SNPs, whereas the Wald ratio test was used for analyzing samples with only one SNP (29).

For sensitivity analyses, First, we calculate the False Discovery Rate (FDR)-corrected p-value, which effectively reduces the risk of false positive results that arise from multiple comparisons. The formula for FDR calculation is FDR = P-value * Rank(max)/P(Rank), where Rank(max) is the total number of exposures included in the study, and P(Rank) is the ranking order of each exposure’s p-value with respect to the outcome MR results. heterogeneity was measured using the Cochran Q method. In case of obvious heterogeneity (p < 0.05), MR-Egger regression analysis was applied to assess the potential pleiotropic inheritance of the SNPs used as IVs. In MR-Egger regression, the intercept term indicates directed horizontal pleiotropy at p < 0.05. Finally, we further tested the stability of the results using multivariable Mendelian randomization and reverse Mendelian randomization.

Statistical analyses were performed using the R package in the R language application (v4.2.1).

## 3 Results

### 3.1 MR analysis of immune cells and lung cancer

**Figure 2:**
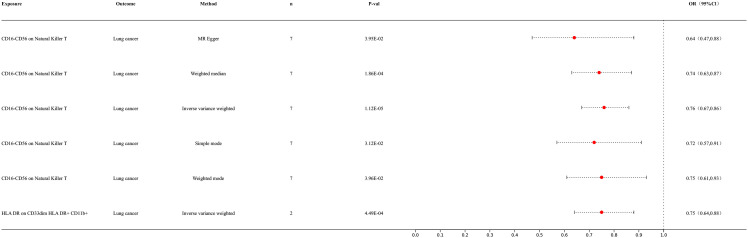
Forest map of Mendelian randomization (MR) results of immune cells and lung cancer.

Our preliminary research results indicated a potential causal relationship between two types of immune cells and lung cancer (**Figure 2**). The IVW analysis results for the classification of these two immune cells were as follows: CD16-CD56 on NK T cells [p = 1.12E-05; OR 95% confidence interval [CI] = 0.76 (0.67, 0.86)]; HLA DR on CD33dim HLA DR+ CD11b+ cells [p = 4.51E-05; OR 95% CI = 0.76 (0.67, 0.86)]. Our results indicated a negative correlation between immune cell infiltration and lung cancer, suggesting that the risk of lung cancer decreases with an increase in the levels of these immune cells. For detailed information on other immune cell results in the MR analysis of immune cells and cancer, please refer to the **Supplementary Materials** (**Supplementary Table S1**).

### 3.2 MR analysis of immune cells and inflammatory factors

**Figure 3:**
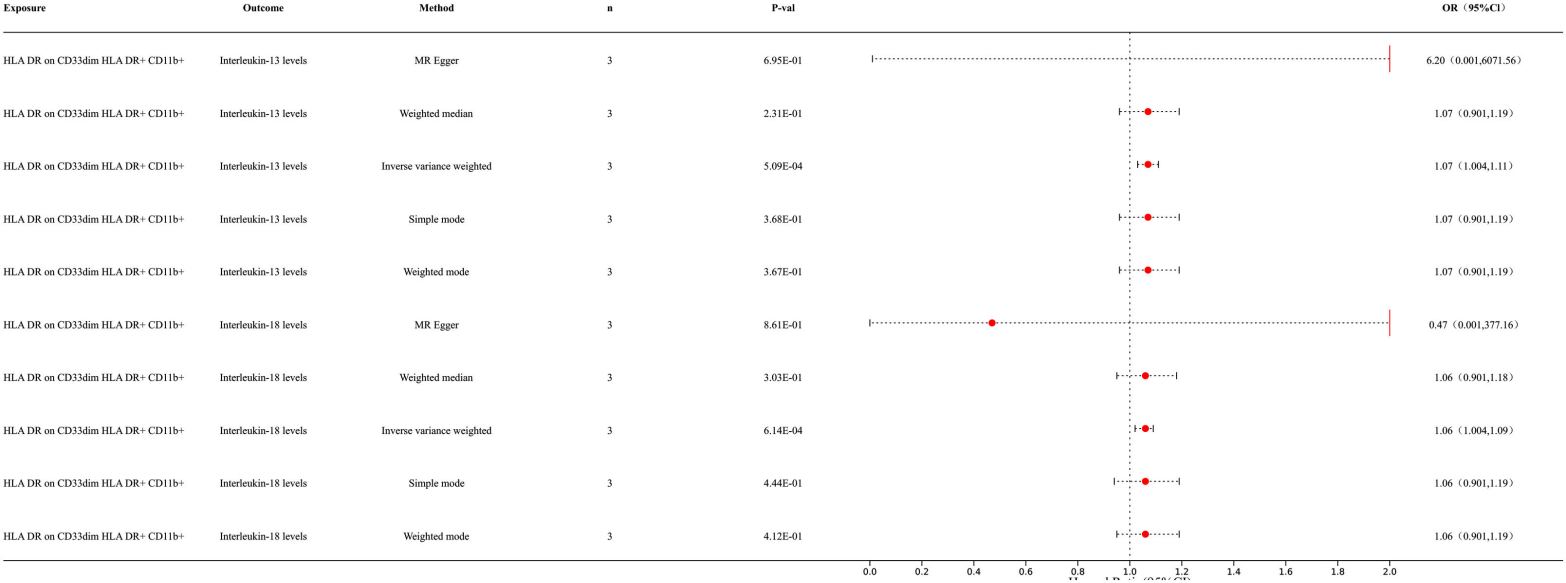
Forest map of MR results of immune cells and inflammatory factors.

Our research results indicate a causal relationship between two types of inflammatory factors and immune cells (**Figure 3**). The results of IVW analysis for the relationship between immune cells and inflammatory factors are as follows: HLA DR on CD33dim HLA DR+ CD11b+ cells and interleukin (IL)-18 levels [p = 6.14E-04; OR 95% CI = 1.06 (1.02, 1.09)]. HLA DR on CD33dim HLA DR+ CD11b+ cells and IL-13 levels [p = 5.09E-04; OR 95% CI = 1.07 (1.03, 1.11)]. Our results indicated a positive correlation between immune cells and inflammatory factors. For detailed information, please refer to the **Supplementary Materials** (**Supplementary Table S2**).

### 3.3 MR analysis of inflammatory factors and lung cancer

**Figure 4:**
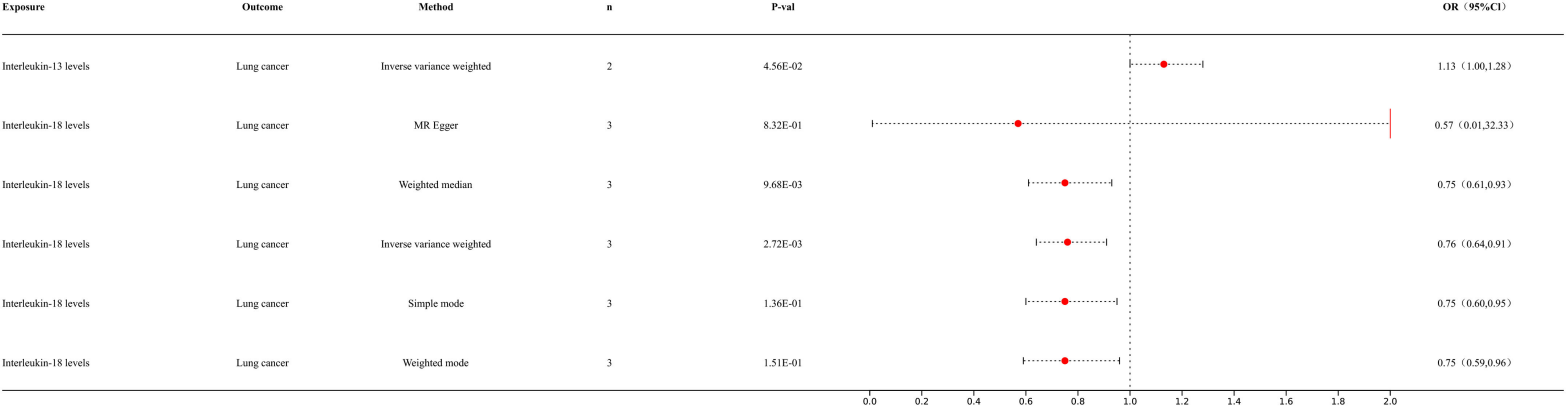
Forest map of MR results of inflammatory factors and lung cancer.

We conducted MR analysis of inflammatory factors and lung cancer to further elucidate the interactions among immune cells, inflammatory factors, and lung cancer (**Figure 4**). Our IVW analysis results showed that IL-18 levels provide the following outcome for lung cancer (p = 2.72E-03; OR 95% CI = 0.76 (0.64, 0.91)). The IL-13 levels provided the following outcome for lung cancer (p = 4.56E-02; OR 95% CI = 1.13 (1.00, 1.28)). The IL-5 levels provided the following outcome for lung cancer (p = 2.49E-02; OR 95% CI = 1.46 (1.05, 2.03)). For detailed information, please refer to the **Supplementary Materials** (**Supplementary Table S3**).

In summary, our study identified two axes in the immune cell-inflammatory factor-lung cancer pathway, namely HLA DR on CD33dim HLA DR+ CD11b+ - Interleukin-18 - lung cancer and HLA DR on CD33dim HLA DR+ CD11b+ - Interleukin-13 - lung cancer. Subsequently, we calculated the mediating effects of these two axes, and found that the mediating effects of Interleukin-13 and Interleukin-18 in these two axes were 7.0% and 6.0%, respectively. The results are presented in **Table 1**.

**Table 1:** Detailed information on the immune cell-inflammatory factor-lung cancer axis.

Exposure	Mediating factor	Outcome	Mediation effect
HLA DR on CD33dim HLA DR+ CD11b+	Interleukin-13	Lung cancer	0.07
HLA DR on CD33dim HLA DR+ CD11b+	Interleukin-18	Lung cancer	0.06

### 3.4 Multivariable Mendelian randomization

**Figure 5:**
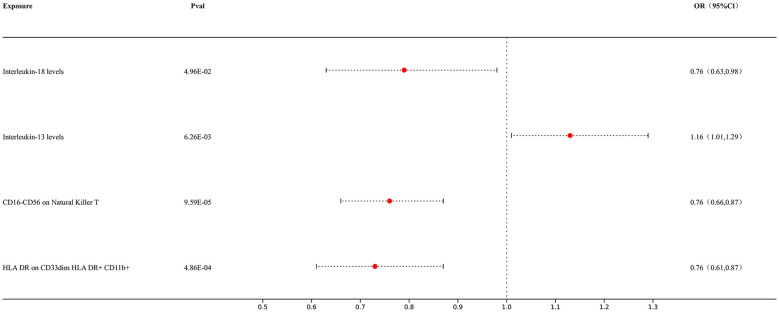
Forest map of MVMR.

In our MVMR study, we conducted MVMR analyses on immune cells and positive target exposures in the analysis of inflammatory factors MR with lung cancer to further ascertain their outcomes. Our results showed causal relationships between immune cells and inflammatory factors: Interleukin-18 levels exhibited a significant association with lung cancer risk (p = 4.96E-02; OR 95% CI = 0.76 [0.63, 0.98]), Interleukin-13 levels were also significantly associated (p = 6.26E-03; OR 95% CI = 1.16 [1.01, 1.29]), CD16-CD56 expression on Natural Killer T cells demonstrated a significant inverse association (p = 9.59E-05; OR 95% CI = 0.76 [0.66, 0.87]), and HLA DR expression on CD33dim HLA DR+ CD11b+ cells showed a significant inverse relationship with lung cancer risk (p = 4.86E-04; OR 95% CI = 0.76 [0.61, 0.87]). The outcomes are presented in **Figure 5**.

### 3.5 Reverse Mendelian randomization and sensitivity analysis

In our reverse MR analysis, our results did not indicate a causal relationship between lung cancer and immune cells, inflammatory factors, or the interaction between inflammatory factors and immune cells. This suggests that our MR findings were not influenced by reverse causation. The specific results are as follows: Lung cancer and Interleukin-18 levels (p = 0.39; OR 95% CI = 1.14 (0.89, 1.29)), Lung cancer and Interleukin-13 levels (p = 0.65; OR 95% CI = 0.65 (0.47, 1.01)), Lung cancer and CD16-CD56 on Natural Killer T cells (p = 0.160; OR 95% CI = 1.21 (0.87, 1.31)), Lung cancer and HLA DR on CD33dim HLA DR+ CD11b+ cells (p = 0.43; OR 95% CI = 1.36 (0.89, 1.81)), HLA DR on CD33dim HLA DR+ CD11b+ cells and Interleukin-18 levels (p = 0.31E; OR 95% CI = 1.36 (0.96, 1.81)), HLA DR on CD33dim HLA DR+ CD11b+ cells and Interleukin-13 levels (p = 0.89; OR 95% CI = 1.16 (0.95, 1.81)).

We subsequently conducted sensitivity analysis to further validate our results. Both heterogeneity and multiplicity tests showed p-values > 0.05, indicating no anomalies in the results. For detailed information, please refer to the **Supplementary Materials** (**Supplementary Tables S4**-**5**).

## 4 Discussion

Based on a large body of publicly available genetic data, we explored causal associations among 731 immune cell traits, inflammatory factors, and lung cancer. To the best of our knowledge, this is the first MR analysis to explore the causal relationship between multiple immune phenotypes and lung cancer. The results of this study showed that two immune phenotypes had a significant causal relationship with lung cancer (FDR < 0.05), with two inflammatory factors playing a significant role in the relationship between immune cells and lung cancer.

Our results indicated a negative correlation between HLA DR expression on CD33dim HLA DR+ CD11b+ cells and lung cancer, i.e., the risk of lung cancer decreases with an increase in HLA DR expression on CD33dim HLA DR+ CD11b+ cells, an immunophenotype indicating the expression of both CD33 and CD11b on the cell surface of certain myeloid cells. In some cases, the CD33dim+ CD11b+ subpopulation may play an important role in inflammatory responses, immunomodulation, and disease development (30, 31). They may be involved in regulating immune functions, such as the activation of immune cells, phagocytosis of pathogens, and modulation of inflammatory responses. In addition, IL-13 and IL-18 play an important role in the relationship between HLA DR expression on CD33dim HLA DR+ CD11b+ cells and lung cancer, and IL-18 plays an important role in lung cancer.

IL-18 is a pro-inflammatory cytokine that is produced by various cells, such as monocytes, macrophages, and epithelial cells (32). Studies have revealed that IL-18 can be consistently and effectively expressed in A549 human lung cancer cells, leading to the inhibition of cell proliferation and tumor cell growth, as well as the promotion of tumor cell apoptosis. Moreover, IL-18 expression triggers the secretion of FN-γ while reducing IL-4 production, thereby restoring the balance between Th1/Th2 cell subsets and highlighting the anti-tumor potential of IL-18 (33). Additionally, research indicates that IL-18 plays a crucial role in enhancing immune responses and positively influencing immunotherapy for lung cancer by modulating the expansion and phenotypic changes of NK cells. These NK cells activated by IL-18 demonstrate accelerated proliferation rates and heightened anti-tumor capabilities, including cytotoxicity, antibody-dependent cytotoxicity, and cytokine production, ultimately exerting anti-tumor effects (34, 35). In the context of lung cancer, IL-18 serves diverse functions. It can stimulate NK cell activity, augmenting their tumor-killing abilities, and also enhance T cell activation and proliferation to bolster anti-tumor immune responses (35, 36). It can induce an inflammatory response, leading to an increase in the infiltration of inflammatory cells, potentially promoting tumor growth and metastasis (37). Additionally, it can promote angiogenesis by stimulating the proliferation of vascular endothelial cells, providing nutrients and oxygen to tumors, and aiding in their growth and spread. Moreover, IL-18 can regulate the expression and activity of apoptosis-related proteins, potentially promoting apoptosis and inhibiting tumor growth (32, 38). Overall, the effects of IL-18 in lung cancer are complex and involve immune response promotion, tumor growth inhibition, and inflammatory response and angiogenesis promotion, which eventually affect tumor development (39, 40). The specific mechanism of action and effects of IL-18 may be influenced by various factors, including tumor type, tumor microenvironment, and immune status. This necessitates further studies that will help understand its role in lung cancer.

IL-13, a cytokine that regulates various cellular functions and immune responses by binding to its receptor, exhibits a dual role in lung cancer, inhibiting apoptosis and promoting tumor cell proliferation (41). In non-small cell lung cancer (NSCLC), the expression levels of IL-13 have been found to be increased in all histological subtypes, with particularly elevated expression in squamous cell carcinoma (SCC) compared to large cell carcinoma (LCC). LCC exhibits an aggressive phenotype, often growing rapidly and spreading faster than other NSCLC subtypes. Therefore, the differential expression of IL-13 in SCC and LCC may be valuable for the clinical practice of NSCLC (42, 43). The adverse evidence of IL-13 in lung cancer primarily lies in its association with tumor development and prognosis. Despite the increased expression of IL-13 in NSCLC, its specific role and impact remain unclear. Some studies suggest that IL-13 is associated with invasion, metastasis, and poor prognosis in other human epithelial cancers. However, for lung cancer, the role of IL-13 has not been fully elucidated. Therefore, our research provides partial evidence for the adverse prognosis of IL-13 in lung cancer (44–46). On one hand, IL-13 may exert its effects by inhibiting tumor cell apoptosis through the activation of intracellular signaling pathways when it binds to receptors on the surface of tumor cells (47). potentially promoting the growth and survival of tumor cells. On the other hand, it can also inhibit tumor cell growth by activating specific and non-specific anti-tumor defense mechanisms. IL-13 enhances the activity of tumor-specific cytotoxic T-lymphocytes (CTLs) and promotes their disruptive effect on tumor cells (48). Furthermore, it enhances the production of tumor-specific interferon gamma (IFN-γ), further bolstering the anti-tumor immune response. However, despite the dual role of IL-13, inhibiting its activity can inhibit the growth and spread of tumor cells (49), making IL-13 a potential target for lung cancer treatment. In some studies, antibodies or antagonists against IL-13 or its receptor are being developed for this purpose.

IL-13 and IL-18 play complex and diverse roles in the development of cancer, involving not only tumor growth, dissemination, and immune evasion mechanisms but also providing new targets for cancer prognostic biomarkers and therapeutic interventions (50–52). With the advancements in immunotherapy and precision medicine, therapeutic strategies targeting these cytokines—including IL-13-targeted toxin fusion proteins, anti-IL-13 antibodies, small molecule drugs that block IL-13 and its receptor interaction, as well as leveraging IL-18’s immune-enhancing properties or enhancing anti-tumor immune recognition and killing through gene therapy—have shown potential in inhibiting tumor growth and spread (38, 53, 54). However, given the intricate role of these cytokines in oncology, the development of these therapeutic strategies requires deeper research to understand their precise mechanisms and to balance activating the immune system with avoiding the promotion of tumor growth when designing safe and effective treatment plans. Therefore, the study and application of these cytokines continue to represent significant potential and challenges in future cancer treatments.

The advantage of this study compared to previous research lies in the fact that other studies have only used two-sample Mendelian randomization to separately investigate the relationships between immune cells and lung cancer, or between inflammatory factors and lung cancer. This study, however, utilized a mediation Mendelian randomization approach to explore the causal relationship within the immune cell-inflammatory factor-lung cancer axis, providing a more precise inquiry for immunotherapy in lung cancer. Our analysis offers significant reference points for understanding the mechanisms of cancer development and designing immunotherapeutic strategies. However, a limitation of this study is that the samples solely come from European populations, which may restrict the generalizability of the research findings across a broader demographic. Further investigation is necessary to address this limitation.

## Conclusion

This study explored the causal relationship among immune cells, inflammatory factors, and lung cancer using the MR method. The results indicate a potential negative correlation between the infiltration of certain immune cells and lung cancer and also point to a positive correlation between inflammatory factors and immune cell infiltration. Specifically, the expression of CD16-CD56 on NK T cells and HLA DR on CD33dim HLA DR+ CD11b+ cells exhibited a negative correlation with lung cancer, whereas the expression of HLA DR on CD33dim HLA DR+ CD11b+ cells was positively correlated with IL-18 and IL-13 levels. Additionally, IL-18 levels were negatively correlated with lung cancer, whereas IL-13 levels were positively correlated with lung cancer. These findings provide an important reference point for understanding the mechanisms underlying lung cancer development and designing immunotherapy strategies. Overall, this study provides a novel theoretical basis for the prevention and treatment of lung cancer, offering valuable insights for future research and the clinical treatment of lung cancer.

## Data Availability

The data used in the present study are all publicly available at https://gwas.mrcieu.ac.uk/, further inquiries can be directed to the corresponding author/s.

## Supplementary material

Supplementary Table S1 All immune cells and lung cancer MR results.

Supplementary Table S2 All Inflammatory factors and lung cancer MR results.

Supplementary Table S3 All immune cells and Inflammatory factors MR results.

Supplementary Table S4 All heterogeneity tests.

Supplementary Table S5 All multiple testing corrections.
